# Evaluating the advancements and efficacies in pharmacological Mpox treatments: a comprehensive review

**DOI:** 10.3389/fphar.2025.1654467

**Published:** 2025-09-30

**Authors:** Shuaibu Abdullahi Hudu, Najlaa Saadi, Albashir Tahir, Abdulgafar Olayiwola Jimoh, Aliyu Haruna

**Affiliations:** ^1^ Department of Microbiology, College of Medicine, Northern Border University, Arar, Saudi Arabia; ^2^ Center for Health Research, Northern Border University, Arar, Saudi Arabia; ^3^ Department of Basic and Clinical Medical Sciences, Faculty of Dentistry, Zarqa University, Zarqa, Jordan; ^4^ Department of Pharmacology, Faculty of Basic Medical Sciences, Bauchi State University, Gadau, Nigeria; ^5^ Department of Pharmacology and Therapeutics, Faculty of Basic Clinical Sciences, College of Health Sciences, Usmanu Danfodiyo University, Sokoto, Nigeria; ^6^ Department of Internal Medicine, Federal Medical Centre Gusau, Gusau, Nigeria; ^7^ Department of Public Health, Iconic Open University, Sokoto, Nigeria

**Keywords:** mpox, tecovirimat, pharmacological therapy, drug resistance, outbreak preparedness

## Abstract

Monkeypox, now known as Mpox, has reemerged as a serious public health threat due to an increasing number of outbreaks outside its primary endemic regions. Although virologically similar to smallpox, smallpox therapy is not specifically approved for Mpox. The goal of this review is to assess the pharmacological progress and therapeutic efficacy of available and new therapies for Mpox. Structured literature review methodology was used based on peer-reviewed articles, clinical trials data and global health agency reports published from 2008 to 2025. Data was collected from ClinicalTrials.gov, Scopus and WHO databases with keywords on antiviral pharmacodynamics, resistance mechanism and clinical outcome. Prominent reviewed stewards include tecovirimat, brincidofovir, and cidofovir: drugs first developed for smallpox but repositioned for Mpox under expanded access programs. Tecovirimat seems most promising, however endangered by emerging resistance mutations. Innovative strategies, including mRNA vaccines, use of nanoparticles for drug delivery and host-directed treatments, are discussed that may improve treatment efficacy and preparedness for outbreaks. The results underscore that combination regimens will be required to counter resistance, there is a need for increased access in low- and middle-income countries, and global health cooperation should be bolstered. The review endorses increased clinical trial capacities, amended regulatory approaches development of new classes of therapeutics as part of global Mpox response efforts. By combining pharmacological breakthroughs with public health readiness, global health communities can be better equipped to respond to Mpox and other possible orthopox outbreaks of the future.

## 1 Introduction

Mpox, better known as monkeypox, is a viral zoonotic disease caused by the monkeypox virus (MPXV), an orthopoxvirus in the family Poxviridae. It also includes the variola virus (responsible for smallpox) and vaccinia virus (used in smallpox vaccines). Mpox was first detected in primates and monkeys kept in Denmark in 1958, thus the name “monkeypox.” The human case was first identified in the year 1970 (Pittman et al., 2022), namely in the Democratic Republic of Congo (DRC), during a time of heightened smallpox eradication efforts. Mpox is mostly spread to people through close contact with infected animals, including rodents and primates, or by eating bushmeat. Human transmission is possible through respiratory droplets, direct contact with faeces, vomiting, or other bodily fluids, or contaminated materials (Amer et al., 2023). The disease is endemic in Central and West Africa, and sporadic outbreaks can occur outside these regions through international travel or imported animals. Until recently, Mpox has been a less common disease of small public health significance and outbreaks compared to other diseases, but interest in Mpox has focused on its index case: close similarity to the smallpox virus, a viral pathogen that was eradicated in 1980. The decline in routine vaccination coverage against smallpox, which conferred some protection against Mpox, has led to increased susceptibility of populations to the virus (Rimoin et al., 2010). The symptoms of MPox are like but less severe than those of smallpox. It usually starts with fever, headache, muscle aches and lymphadenopathy (swollen lymph nodes), and then progresses into macules, papules, vesicles, pustules and scabs. The duration of the acute illness is typically 2–4 weeks, and case fatality rates vary depending on viral clade and access to healthcare, ranging from 1% to 10% (Og et al., 2023).

Mpox viruses are genetically divided into two major clades, formerly termed the Central African (Congo Basin) clade and the West African clade, but recently reclassified by the WHO as Clade I and Clade II, respectively (Karagoz et al., 2023). Clade I, historically associated with the Congo Basin region, has shown higher transmissibility, more severe clinical manifestations, and case fatality rates approaching 10%, particularly in populations with limited healthcare access (Karagoz et al., 2023). Clade II, predominant in West Africa and responsible for the 2022 multi-country outbreak, is further subdivided into subclades IIa and IIb, the latter including the outbreak lineage. This clade generally causes milder disease with lower mortality (<1%), but can still produce severe illness in immunocompromised individuals, children, and pregnant women (Karagoz et al., 2023; Cevik et al., 2024). The wider potential of Mpox to spread, especially beyond endemic regions, has made it globally relevant. In 2022, a multi-country outbreak of Mpox was observed, with cases detected in Europe, North America, and other regions for the first time, clearly showing sustained human-to-human transmission occurring outside Africa (Rehan et al., 2023). This outbreak showed us that we need better surveillance, diagnostics, and public health preparedness to address emerging infectious diseases. Mpox also highlights the need for One Health approaches, which acknowledge relationships among human, animal and environmental health (Abd ElHafeez et al., 2023). Mpox serves as a relevant case study towards mitigating future pandemics, some of which may be zoonotic in cause, due to increased risk of zoonotic spillover because of deforestation, wildlife trade, or climate change (Jones et al., 2008).

Developing therapeutic agents is critically important in managing and controlling Mpox, especially considering its outbreak potential and the lack of specific antiviral therapy for the disease. Pharmacological interventions are critical to decreasing morbidity, mortality and transmission rates and thus contain the overall public health impact of Mpox. Unlike smallpox, which comes with well-developed treatments, no specific antiviral therapies exist for Mpox. Current treatment options include repurposed antivirals that were initially developed for smallpox or other viral infections, the most notable of these being Tecovirimat (TPOXX), Brincidofovir, and Cidofovir (Huston et al., 2023; Shah and Modi, 2024). These medications block viral replication but require further studies to optimize their efficacy and safety for Mpox. Finally, there are no antiviral drugs approved for Mpox, and efforts should be made to develop Mpox-specific antivirals targeting the mechanisms specific to the monkeypox virus, which could be translated into effective treatments. Mpox can lead to severe disease,including painful skin lesions, secondary bacterial infections and systemic complications. Drugs tackle symptoms, help shorten illness duration, and prevent complications, e.g., the development of sepsis or pneumonia (Ramírez-Soto and Arroyo-Hernández, 2024). Administering antivirals at the early phases of viral infection could help to limit viral replication and reduce disease severity in susceptible populations, including individuals who are immunocompromised, children, and pregnant women. Some effective pharmacological therapies can also lower the number of viruses in infected individuals, hence reducing the risk of human-to-human transmission. Combining antivirals with vaccination and isolation is an important component of a broader public health strategy to help control the spread of Mpox within our communities. Although vaccines like JYNNEOS (Imvamune/Imvanex) and ACAM2000 are valuable for preventing Mpox, access is inconsistent, and distribution can be logistically difficult, particularly in low-resource environments (Rimoin et al., 2010).

Pharmacological treatments present an alternative or complementary approach to vaccination in these individuals who cannot receive the vaccines due to contraindications, e.g., immunocompromised individuals, and in post-exposure prophylaxis. Also, like other viruses, the monkeypox virus may evolve resistance to currently available antivirals. Jones et al. (2008) point to the need for advancing pharmacological approaches to discover new pharmacological agents as well as combination therapy to avoid resistance and maintain long-term treatment efficacy (Jones et al., 2008). Mpox disproportionately impacts low- and middle-income countries (LMICs), primarily in Central and West Africa, where health systems are weak. This gap can be remedied with innovative pharmacological tools, providing low-cost, accessible, and effective tools for vulnerable populations (Ofori et al., 2024). Research and development investments can also enable the production of generic versions of antivirals for wider availability in resource-limited settings. The global Mpox outbreak in 2022 has shown us that we need to be prepared for emerging infectious diseases. Developments in pharmacology, such as broad-spectrum antivirals, may also better prepare the world to respond to future outbreaks of Mpox or other orthopoxviruses (Malik et al., 2023). Findings from research on Mpox treatments may also inform the fight against other zoonotic diseases, adding to the body of knowledge of the pathogenesis of viruses and potential therapeutic targets. Mpox outbreaks can wreak havoc on healthcare systems and cause exorbitant direct and indirect economic costs from hospitalisation, lost productivity and public health measures. Effective pharmacological treatments can relieve the burden on healthcare systems and reduce the socioeconomic impact of outbreaks (Jones et al., 2008). Research and development are critical areas of global health that can help us respond more effectively to Mpox outbreaks, as well as prepare for the next antimicrobial-resistant infectious disease and public health emergencies affecting health equity globally.

The review article reiterates the critical state of pharmacological advancements for treating Mpox elemental for the fact that there is no approved specific antiviral for the treatment of this orthopoxvirus, and only repurposed drugs, e.g., tecovirimat, brincidofovir, and cidofovir, are available (Ezat et al., 2023). It highlights the requirement of optimized treatments to diminish disease severity, prevent complications, and control transmission, especially in outbreak settings. The review discusses limitations of available vaccines and potential pharmacological alternatives in resource-limited settings and high-risk populations (Ezat et al., 2023). It also examines the potential for antiviral resistance and the need for the development of new drugs to maintain long-term efficacy (Jones et al., 2008). Improving pharmacology can help promote global health equity by improving outbreak preparedness and reducing the socioeconomic burden of Mpox.

## 2 Methodology

This review was conducted as a structured narrative literature review to consider both established and emerging pharmacological therapies for Mpox. Systematic search for relevant studies was conducted in PubMed, Scopus, Web of Science, ClinicalTrials.gov and the WHO Global Research Database. The search was specifically designed to capture literature published between 2008 and 2025 with search term combinations, such as “Mpox treatment,” “monkeypox antiviral,” “tecovirimat efficacy,” “drug resistance,” and “emerging therapeutics.” The selection criteria were peer-reviewed original research articles, clinical trials, meta-analyses, case reports, and official policy documents. More grey literature and technical reports from the CDC, Africa CDC, and WHO were added to be more inclusive of the real-world practice, emergency use authorisations, and therapeutic recommendations. Articles were filtered using the criteria of the pharmacological activity, mechanism of action, resistance profile, clinical outcome and public health risk associated with treatment for Mpox. Data was methodologically critiqued and appraised for coherence with the identified themes. During the synthesis, we compared the advantages and disadvantages of each intervention, with special attention to their potential for use in complementary high- and low-resource settings. This approach allowed for a detailed overview of the current treatment situation and determination of potential shortcomings and future research needs.

## 3 Current therapeutic strategies

### 3.1 Overview of existing antiviral medications

A variety of approaches for prevention and treatment have been developed to counter Mpox, encompassing the ACAM2000 and MVA-BN7 vaccines, the immunoglobulin VIGIV, and a variety of antiviral drugs such as Tecovirimat, Brincidofovir, and Cidofovir. These medications play an important role in controlling severe manifestations of the infection (Chenchula et al., 2023), especially in immunocompromised patients, ensuring better management and reducing its damage (Okesanya et al., 2025). Given the growing cases of Mpox infection worldwide, evaluating the feasibility of treatment strategies to control potential outbreaks has become important (Okesanya et al., 2025). Tecovirimat is an effective antiviral medication used to take measures to prevent and control smallpox and other Orthopoxvirus-related illnesses (Okesanya et al., 2025). It is used to stop the spread of the virus from infected cells (DeLaurentis et al., 2022; Falasca et al., 2025). Its effectiveness has been confirmed through extensive research in animal models and a restricted number of human cases (DeLaurentis et al., 2022; Falasca et al., 2025). Current guidelines recommend Tecovirimat for adults and pediatric patients weighing ≥40 kg at a dose of 600 mg orally every 12 h for 14 days, administered within 30 min after a moderate-to high-fat meal to enhance bioavailability. For pediatric patients weighing 13–<25 kg, the suggested dose is 200 mg every 12 h; for those weighing 25–<40 kg, the dose is 400 mg every 12 h. An intravenous formulation is available for patients unable to take oral medication: 6 mg/kg every 12 h for individuals ≥2 years old and weighing <120 kg, or 200 mg every 12 h for those ≥120 kg, infused over at least 6 h to reduce infusion-related hypotension risk (DeLaurentis et al., 2022; Mishra et al., 2025; Bogacka et al., 2025; Siegrist and Sassine, 2023). The usual course is 14 days for uncomplicated disease, with consideration for prolonged therapy in severely immunocompromised patients or those with delayed lesion healing. While no dosage adjustments are required for renal impairment with the oral form, caution is advised for the IV formulation in moderate-to-severe renal dysfunction due to the hydroxypropyl-β-cyclodextrin excipient (Rao et al., 2023).

Tecovirimat, also identified as ST-246, has received approval from the United States Food and Drug Administration (US FDA) for the treatment of smallpox infections caused by the variola virus. This antiviral medication has been officially approved for use in both adult and pediatric patients. While primarily approved by the FDA for smallpox treatment, it is also utilized in an off-label capacity to address Mpox infections (Chenchula et al., 2023; Russo et al., 2023). Additional antivirals include Brincidofovir, a prodrug of Cidofovir, as a pharmaceutical preparation for oral route of drug administration, which prevents viral DNA polymerase activity. It has been utilised in certain Mpox cases as another treatment when Tecovirimat is unavailable (Siegrist and Sassine, 2023; Huston and Egelund, 2025). An alternative effective antiviral agent is Cidofovir, which impairs viral DNA replication by DNA polymerase suppression. It has been employed in life-threatening Mpox cases, although its application is limited due to kidney toxicity (Siegrist and Sassine, 2023; Bruno and Buccoliero, 2023).

Compared to Tecovirimat, both Cidofovir and Brincidofovir present notable safety concerns that influence their clinical use. The FDA and EMA highlight nephrotoxicity as the major limiting factor for Cidofovir; it can cause dose-dependent proximal tubular damage, potentially leading to acute renal failure, particularly in patients with preexisting kidney impairment (Dobrek, 2023; Liu et al., 2025). Consequently, Cidofovir administration requires concurrent probenecid and intravenous hydration, which can be logistically challenging in outbreak or low-resource settings (Wolf et al., 2003). Other reported adverse effects include neutropenia, ocular hypotony, and metabolic acidosis (Upadhyayula and Michaels, 2013). Brincidofovir, a lipid-conjugated prodrug of Cidofovir developed to improve bioavailability and reduce renal toxicity, has its own distinct safety profile (Imran et al., 2023). Studies have noted a risk of hepatotoxicity, with significant elevations in serum transaminases and bilirubin observed in some patients, occasionally leading to treatment discontinuation. Gastrointestinal disturbances, such as diarrhea and nausea, are also common (Imran et al., 2023). In contrast, Tecovirimat has demonstrated a more favorable safety profile in both animal models and human use, with most adverse effects limited to mild headache, nausea, and transient gastrointestinal symptoms (Okesanya et al., 2025). It does not require renal protective measures or have significant hepatotoxicity in current clinical experience. This comparatively benign safety profile, combined with oral dosing convenience and fewer monitoring requirements, underscores the importance of Tecovirimat as the preferred first-line antiviral for Mpox where available, reserving Cidofovir and Brincidofovir for cases where Tecovirimat is contraindicated or unavailable.

### 3.2 Emergency use authorizations (EUAs) and their impact

Starting in 2012, the U.S. Centres for Disease Control and Prevention (CDC) has assisted access to Tecovirimat *via* an Expanded Access Investigational New Drug Expanded Access IND (EA-IND) program, enhancing its application outside of smallpox treatment. This guideline facilitates healthcare providers administering Tecovirimat for uncommon orthopoxviral infections, as well as laboratory incidents involving Vaccinia virus and serious difficulties from live, replication-competent smallpox vaccines. By allowing off-label use in these specific applications, the program services mitigate risks related to unexpected exposure and undesirable vaccine effects (Yu et al., 2024). Concerning the expanded use and global initiatives, Tecovirimat has been authorized for humanitarian use protocols for treating complex patients of vaccinia and cowpox, with no major safety issues described. Meanwhile, projects are advancing to develop an extended access program in the Central African Republic, an area that continues to experience repeated Mpox outbreaks (Lindholm et al., 2019; Vora et al., 2008). Between May 2022 and July 2023, Tecovirimat observed broad use across the United States. Intake forms were sent for more than 7,100 specific cases; however verified count of treated individuals under the EA-IND protocol is likely higher, since certain instances remain unverified (Yu et al., 2024).

### 3.3 Real-world applications and effectiveness

Offering clinical support can aid in relieving the impact of lesions by mitigating additional complications, ensuring suitable hydration and nutrition, and protecting susceptible areas such as the eyes and genital organs. This approach focuses on all-encompassing case management to help recovery and diminish possible risks (Reynolds et al., 2017; Smith et al., 2004). Several studies have evaluated the safety and efficacy of Tecovirimat in treating poxvirus infections. A narrative review examined ten studies, highlighting diverse findings diverse results across diverse patient demographics and study designs. Research involving rabbit and Mpox models highlighted that initial intervention, mainly in exposed groups like People Living with Human Immunodeficiency Virus, assisted in reducing disease complications. While healing periods, symptom alleviation, and viral eradication differed among studies, extended access to Tecovirimat contributed to recovering clinical outcomes with manageable adverse drug reactions. investigational results further confirmed its effectiveness against recent virus strains (Okesanya et al., 2025).

The development of Tecovirimat-resistant Mpox virus was first reported in 2022 among those who had never been introduced to the drug, demonstrating that resistance obtained through Tecovirimat use may have been spread to others. A newly identified infection cluster manifested, in which patients with Tecovirimat-resistant Mpox virus were epidemiologically linked, spanning five states for a 5-month duration (Garrigues et al., 2023; Gigante et al., 2024). A new clinical review studied 25 patients diagnosed with Mpox virus infections, all of whom experienced a 14-day Tecovirimat medication schedule, except for one patient who received 21 days of treatment plan. The study, which modified dosage accordance to patient weight, identified that oral Tecovirimat, dispensed at eight-or 12-h periods, was highly tolerable, with only insignificant adverse drug reactions observed (Desai et al., 2022). Individuals with HIV have experienced severe Mpox virus cases., Tecovirimat has manifested notable therapeutic effectiveness, similar to its effect on HIV-negative individuals struggling against the infection.

Tecovirimat is generally well tolerated, with common adverse events including headache, nausea, abdominal discomfort, and mild elevations in hepatic transaminases (DeLaurentis et al., 2022; Siegrist and Sassine, 2023)). Serious adverse reactions are rare and have not been consistently linked to the drug in clinical use. Data on pregnant women with Mpox are scarce; however, reproductive toxicology studies in rats and rabbits at exposures several-fold higher than the human therapeutic dose revealed no teratogenic effects or adverse pregnancy outcomes (DeLaurentis et al., 2022). Its use during pregnancy should therefore be based on a careful benefit–risk assessment, particularly in cases of severe disease or high-risk exposure. In immunosuppressed populations, including those with advanced HIV infection, observational evidence from the 2022–2023 outbreak indicates that Tecovirimat retains a favorable safety profile and can be clinically effective when initiated early, although disease severity in these groups remains higher due to underlying immune compromise (Ahmed et al., 2023). Preclinical studies in immunocompromised mouse and nonhuman primate models have shown that Tecovirimat maintains antiviral efficacy and survival benefit even in the setting of profound immune suppression, although delayed viral clearance may occur (Russo et al., 2018; Mucker et al., 2013). No Mpox-specific dosing adjustments are currently recommended for these populations; however, close monitoring for clinical progression and potential drug–drug interactions especially with concomitant antiretroviral therapy is advised. Where direct clinical evidence is lacking, these recommendations are supported by smallpox treatment experience and findings from relevant animal models (Siegrist and Sassine, 2023).

This proposes it is possible as a significant treatment choice across different patient populations (McLean et al., 2023). Additional research examined the differences between Brincidofovir and Tecovirimat, the treatment regimen involved Brincidofovir (200 mg orally once per week); however, its use was discontinued after patients exhibited hepatotoxicity due to elevated liver enzyme levels, while the patient received Tecovirimat at a dose of 600 mg orally twice daily for 2 weeks, tolerated well, with no adverse reactions, and exhibited a considerably reduced of illness coarse, hospitalization lasted just 10 days, notably shorter than for the other patients (Adler et al., 2022).

## 4 Novel pharmacological discoveries

### 4.1 Innovative antiviral drugs under development

Viral diseases continue to pose serious challenges for global health. In the domain of chemical pharmacology research, Scientific efforts to discover antiviral drugs must extend beyond persistent infections like AIDS and hepatitis B, which continue to pose challenges to conventional therapies. Instead, scientists should adopt a proactive strategy, prioritizing preparedness for viral outbreaks and advancing the creation of broad-spectrum antiviral treatments to improve preparedness and therapeutic choices (Yao et al., 2021). Progress in the development of novel antiviral treatment approaches is progressing swiftly, aiming to tackle issues such as resistance to treatment, wide-ranging efficacy, and financial accessibility. A key effort is on broad-spectrum antivirals, which are formulated to inhibit diverse viruses simultaneously, reducing the likelihood of drug resistance and broadening treatment options for newly emerging viral threats (Yao et al., 2021). These medications mark a significant step in antiviral treatment strategies, focusing on several wide-ranging antiviral drugs that are being developed to improve treatment choices for several viral infections. Molnupiravir is a broad-spectrum antiviral developed to combat RNA viruses, targeting SARS-**CoV-2** (Elango et al., 2023). In contrast, Remdesivir, initially created for managing the Ebola virus, has been adapted for use in COVID-19 therapy (Chatterjee and Thakur, 2022).

**Paxlovid**, a combination therapy of **Nirmatrelvir and Ritonavir**, is an oral antiviral utilized for treating **COVID-19** infections (Abdelnabi et al., 2022; Rubin, 2024). **Favipiravir** has been investigated for its effectiveness against influenza treatment and emerging viral diseases (Łagocka et al., 2021; Shiraki and Daikoku, 2020; Tsai, 2025)**.** In contrast, Brincidofovir, an improved formulation of Cidofovir, has been studied for its effectiveness against smallpox and monkeypox (Shiraki and Daikoku, 2020). **Lagevrio**, the branded version of **Molnupiravir**, has been employed in **COVID-19** management (Kimata et al., 2023). **Sofosbuvir** is a direct-acting antiviral specifically designed for **hepatitis C** (Xie et al., 2022)**,** while **Baloxavir Marboxil** offers a single-dose solution for influenza (Shirley, 2020). Lefitolimod is under study as an immune-modulating antiviral agent. Lefitolimod administration facilitates the augmentation of CD16^+^ natural killer (NK) cells, which serve a crucial role in antibody-dependent cellular cytotoxicity. CD16^+^ NK cells are essential for detecting and eradicating infected cells, mainly through antibody-dependent mechanisms. Lefitolimod stimulates immunological responsiveness by activating plasmacytoid dendritic cells and B cells, thereby enhancing the body’s capacity to recognize and destroy HIV-infected cells (Gunst et al., 2023)**.** These medications highlight significant progressions in antiviral therapy, emphasizing wide-ranging therapeutic impact, inhibiting the emergence of drug resistance, and innovative solutions for optimizing global health systems (Luong et al., 2025).

### 4.2 Mechanisms of action of new drugs

**FIGURE 1:**
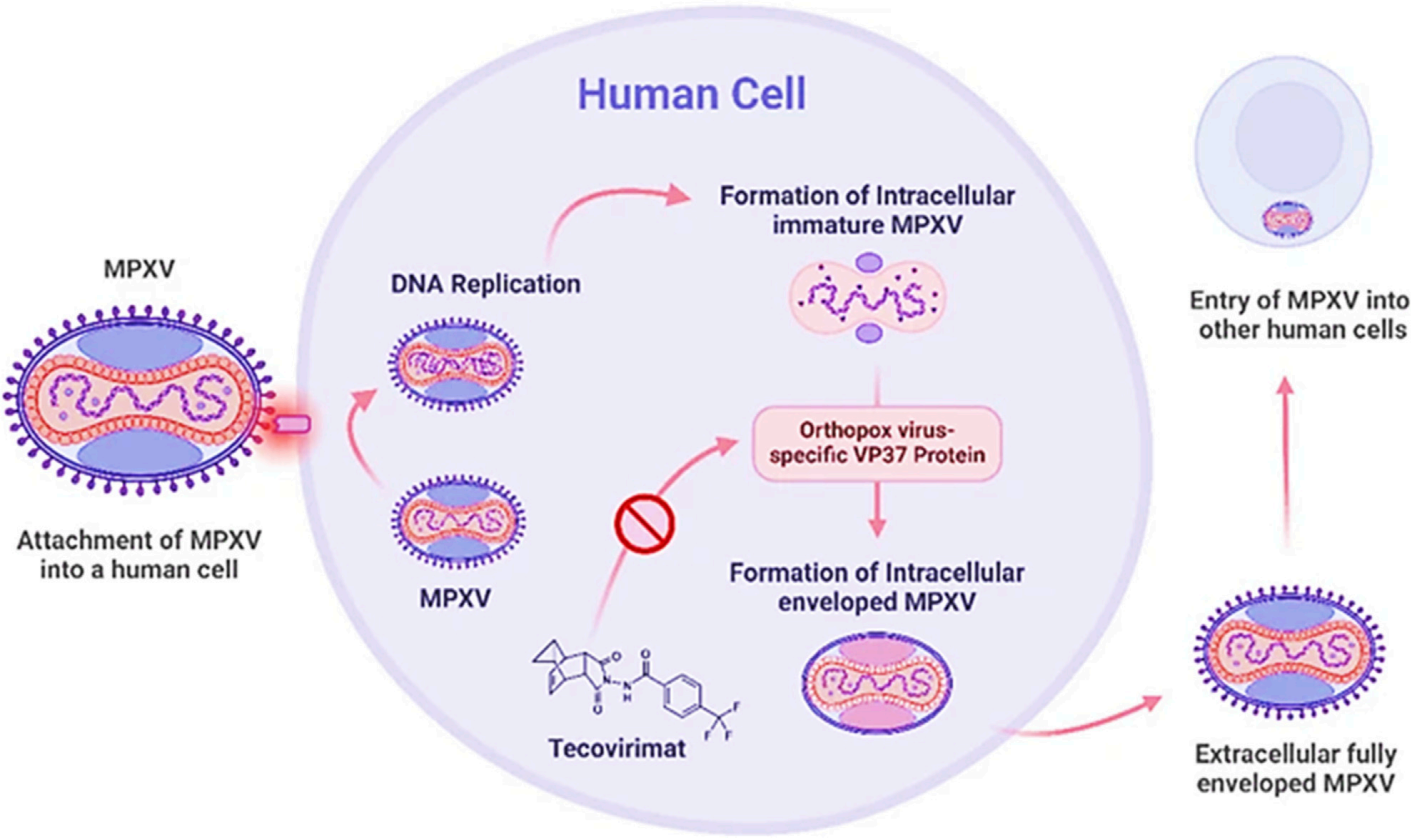
Mechanism of action of Tecovirimat against Mpox (Okesanya et al., 2025).

**FIGURE 2:**
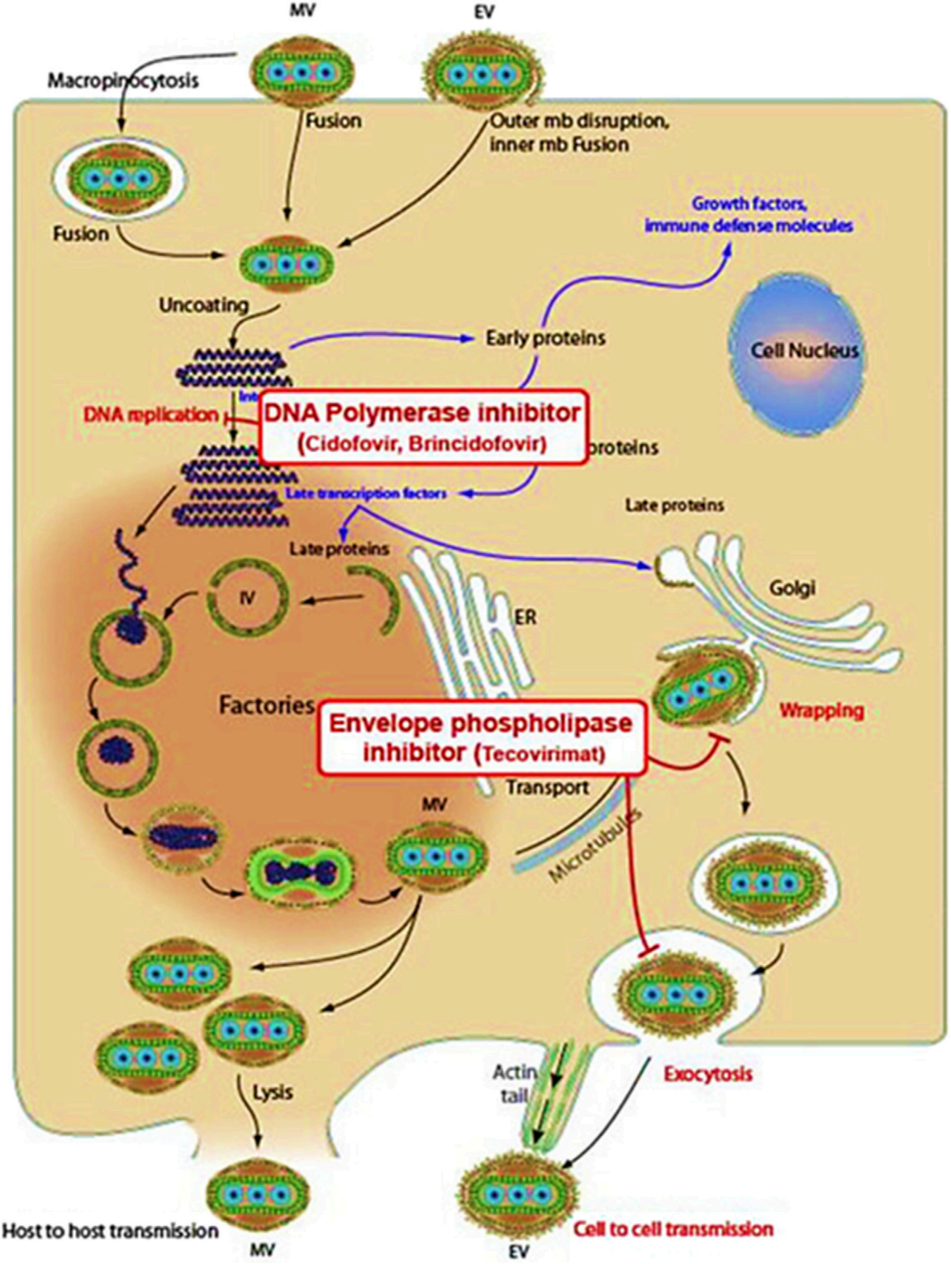
Mechanisms of action of Brincidofovir, Cidofovir, and Tecovirimat against Mpox (Rani et al., 2023).

Tecovirimat inhibits the VP37 protein function, which plays an important role in constructing the viral coat (Okesanya et al., 2025) as shown in Figure 1, by interfering with this protein, the drug effectively stops the virus from creating fully developed enveloped virions, restricting its ability to multiply within the host (Siegrist and Sassine, 2023). Brincidofovir, a modified version of Cidofovir (prodrug), undergoes metabolic activation inside the cell to induce its antiviral efficacy. It selectively interferes with viral DNA polymerase activity, blocking DNA synthesis and disrupting viral replication (Figure 2). It is a lipid-conjugated variant; this formulation enhances bioavailability while minimizing nephrotoxicity associated with Cidofovir (Bruno and Buccoliero, 2023). Cidofovir works as a nucleotide mimic, disrupting viral DNA replication *via* DNA polymerase suppression. By stimulating the enzyme’s natural substrate, it interferes with the synthesis process, inhibiting viral multiplication (Siegrist and Sassine, 2023).

### 4.3 Potential to alter the Mpox treatment landscape

The treatment landscape for Mpox has undergone notable improvements, shaped by ongoing scientific innovation and revolutionary findings. Researchers have progressively introduced new therapeutic methods, revolutionizing disease treatment and enhancing patient health results. Progress in diagnostic technologies has enhanced early detection, enabling early response and more effective disease control. One of the most extensively researched antiviral interventions, Tecovirimat, has shown promising efficacy in restricting viral transmission. The development of mRNA vaccines marks a pivotal shift in preventive medicine, enhancing immune responses to defend against the virus. With ongoing research driving further improvements in treatment approaches, these progresses are strengthening worldwide efforts to combat Mpox more efficiently (Malik et al., 2023).

Regardless of SIGA’s compassionate use donations, through both direct national contributions and distribution *via* the WHO-access to the medication has predominantly been confined to the U.S. and Europe, where it has played a vital role in strategies for responding to national outbreaks. Conversely, the need remains modest across low- and middle-income nations, largely due to a complex interplay of factors. Reduced diagnostic accuracy and insufficient documentation, predominantly in areas with high disease prevalence, have resulted in an underestimation of the disease burden. Furthermore, the less severe manifestation of Mpox in many cases, with lower incidence of hospital admissions and mortality rates, has influenced prioritization of therapeutic interventions. The evolution of Mpox treatment is being shaped by continuous advancements in antiviral medications, diagnostic innovations, and strategies aimed at improving accessibility. Tecovirimat, a notable antiviral, has shown promising effectiveness in mitigating the virus; however, challenges such as inconsistent regulatory policies, insufficient clinical trial data, and concerns surrounding affordability still impact its widespread availability (Ganesan et al., 2025). The continuing exploration of new treatment methods-including synergistic treatment protocols and immune-targeted treatments, facilitates patient recovery. The improvement of rapid diagnostic technologies plays a crucial role in enhancing early detection, enabling early therapeutic action and minimizing viral spread. Mitigating economic and logistical challenges, while strengthening intergovernmental cooperation, pharmaceutical industry producers and scientific research organizations, can enhance the Mpox therapeutic protocols. Integrating groundbreaking healthcare solutions into global disease control frameworks holds a significant opportunity for enhancing the total care approach and reinforcing the global health response to Mpox (Garcia-Atutxa et al., 2024; Zhai et al., 2025).

Tecovirimat is a potent antiviral agent indicated for treating smallpox and additional Orthopoxvirus-associated illnesses. With the escalating prevalence of Mpox cases throughout the world, there is an immediate need to investigate management approaches to control infectious surges (Okesanya et al., 2025). Tecovirimat and Cidofovir demonstrated efficacy for use as post-exposure prophylaxis and clinical management of Mpox. Although their promise, extensive clinical research findings concerning their effectiveness in human Mpox cases remain limited, suggesting the need for additional investigation to confirm their role in therapeutic applications (Bogacka et al., 2025). Currently, there are no specific medications that have official approval for treating Mpox in humans. However, Brincidofovir and Tecovirimat have received certification in the United States for smallpox treatment strategies, chiefly as a defence against potential bioterrorism risks. Regardless of this regulatory approval, neither medication has undergone clinical trials to evaluate therapeutic effectiveness in human Mpox cases. However, both exhibit antiviral activity against orthopoxviruses, including Mpox, in preclinical animal studies, suggesting their potential as therapeutic agents (Adler et al., 2022). Concerning the multi-drug treatment approaches, research indicates synergy between Tecovirimat and Brincidofovir, and using antivirals together with vaccines. Research indicates that combining Tecovirimat and Brincidofovir augments antiviral efficacy both *in vitro* and *in vivo* (Witwit et al., 2025).

## 5 Clinical trials and mechanistic studies

Clinical trials and mechanistic studies on Mpox treatments have yielded critical insights into antiviral efficacy, safety, and future research priorities. The NIH-sponsored STOMP trial found tecovirimat safe but ineffective for mild-to-moderate clade II Mpox. An interim analysis showed no difference in lesion resolution time or pain reduction compared to placebo, leading to early trial termination (CDC, 2025). The MOSA trial in Africa is assessing Brincidofovir’s safety and efficacy, though no results are reported yet. Earlier compassionate-use studies suggested potential benefits, but hepatotoxicity risks remain under investigation (Africa CDC, 2024). A Charité study demonstrated 84% effectiveness for a single Imvanex dose in HIV-negative individuals, but insufficient protection for people with HIV (Raccagni and Nozza, 2025). To further illustrate the scope of completed research, Table 1 summarizes key clinical trials on Mpox, highlighting their study designs, interventions, and outcomes.

**TABLE 1:** Summary of completed clinical trials on Mpox (from the clinicaltrials.gov website).

NCT number	Study title	Interventions	Primary outcome measures	Secondary outcome measures	Study type	Study design	Phase	Start date	Completion date
NCT05443867	Monkeypox Asymptomatic Shedding: Evaluation by Self-Sampling (MPX-ASSESS)	None	Secondary attack rate of MPXV infection in contacts (PCR positivity within 21 days)	Seroconversion rate, asymptomatic infection rate, symptom onset timing	Observational	Prospective	N/A	6/22/2022	8/25/2022
NCT05651581	Efficacy and Acceptability of a Monkeypox Curriculum for Disproportionately Impacted Communities	Online module	Changes in knowledge using a survey	Risk perception, vaccination intention, public health confidence	Interventional	Single-Group Assignment	N/A	5/24/2023	8/24/2023
NCT05543577	Assessing the Preparedness and Knowledge of Pharmacists in the Current Monkeypox Outbreak	Questionnaire	Pharmacist knowledge on monkeypox treatment	Direct interviews on diagnostic skills and attitudes	Observational	Cross-Sectional	N/A	9/14/2022	12/1/2022
NCT00728689	Phase I Trial of an Investigational Smallpox Medication	Drug: ST-246	Pharmacokinetic parameters	Safety and tolerability	Interventional	Randomized Crossover	Phase 1	8/2008	10/2008
NCT05522296	Breakthrough Infection Following Mpox Vaccination	Drug: Mpox Vaccine	PCR-confirmed mpox infection	Symptoms duration, medical treatment requirement, hospitalization, scarring	Observational	Prospective	N/A	9/12/2022	3/30/2024
NCT05476744	Viral Clearance and Epidemiological Characteristics in Patients With Monkeypox	None	Time to undetectable viral load in skin, blood, oropharyngeal swabs	Humoral and cellular immune responses	Observational	Prospective	N/A	6/28/2022	3/30/2023
NCT05559099	Tecovirimat for Treatment of Monkeypox Virus	Drug: Tecovirimat Oral Capsule, Placebo	Time to lesion resolution	PCR negativity, symptom duration, adverse events	Interventional	Randomized Parallel Assignment	Phase 2	10/10/2022	9/3/2024
NCT03745131	Cohort Study of Healthcare Workers Receiving Imvanex^®^	Blood draw	Antibody responses to vaccination	Neutralizing antibodies, adverse events	Observational	Prospective Cohort	N/A	10/30/2018	11/30/2019
NCT05512949	Trial to Evaluate the Immunogenicity of Dose Reduction Strategies of the MVA-BN Monkeypox Vaccine	Biological: JYNNEOS	Neutralization titer at day 43	Adverse events, seroconversion rates	Interventional	Randomized Parallel Assignment	Phase 2	9/9/2022	10/19/2023
NCT05740982	A Phase 2 Randomized Multisite Trial to Inform Public Health Strategies Involving the Use of MVA-BN Vaccine for Mpox	Biological: JYNNEOS	Occurrence of adverse events	Immune response, vaccine efficacy	Interventional	Randomized Parallel Assignment	Phase 2	3/22/2023	8/28/2024
NCT05734508	Assessment of Safety Profile of MVA-BN Vaccine in the PALM-007 Study in DRC	Biological: MVA-BN vaccine	Frequency of serious adverse events	Adverse events, safety profile	Interventional	Single-Group Assignment	Phase 4	3/23/2023	6/28/2024
NCT05976100	Study of the Safety, Tolerability, Pharmacokinetics of NIOCH-14 in Volunteers Aged 18–50 Years	Drug: NIOCH-14	Pharmacokinetic parameters	Safety and tolerability	Interventional	Non-Randomized Parallel Assignment	Phase 1	9/4/2020	3/9/2022
NCT05762523	Safety and Tolerability Study of the VAC∆6 Vaccine in Volunteers Aged 18–40 Years	Biological: VAC∆6 vaccine	Changes in neutralizing antibodies	Safety, immune response	Interventional	Randomized Parallel Assignment	Phase 1	5/18/2019	1/27/2020
NCT05846243	Study on Immunogenicity, Reactogenicity, and Safety of Mpox Vaccine	Biological: Mpox Vaccine	Immunogenicity, antibody response	Adverse events, reactogenicity	Interventional	Randomized Parallel Assignment	Phase 2	4/15/2023	12/20/2023

The studies summarized above provide crucial insights into various aspects of Mpox clinical trials, including asymptomatic shedding, vaccine efficacy, treatment strategies, and public health preparedness. The MPX-ASSESS study (NCT05443867) confirms presymptomatic viral shedding of MPXV, highlighting the risk of transmission during sexual contact. A significant proportion of samples show positive MPXV-PCR results, often with high cycle threshold (Ct) values (Brosius et al., 2023). The findings suggest that asymptomatic infections play a significant role in disease transmission, stressing the need for precautions during the incubation period. Similarly, while specific conclusions were not provided, the study on Mpox vaccination breakthrough infections (NCT05522296) emphasized the persistence of infections despite immunization. Generally, studies on Mpox vaccines like MVA-BN (Jynneos/Imvanex) suggest that while they offer significant protection against Mpox, they do not completely prevent infection, especially after a single dose (Dalton et al., 2023; Guagliardo et al., 2024). On the treatment front, the trial on Tecovirimat (NCT05559099) did not meet its primary endpoint of showing a statistically significant improvement in the time to lesion resolution within 28 days post-randomisation compared to those receiving a placebo. However, a meaningful improvement was observed in patients whose symptoms began 7 days or fewer before randomization and in those with severe disease (defined as having 100 or more skin lesions) (CDC. Mpox, 2025). Additionally, the pharmacokinetic analysis of ST-246 (NCT00728689) provided critical data on drug absorption and metabolism, which could guide future dosing strategies.

Public health preparedness was another key focus. The pharmacist preparedness study (NCT05543577) revealed gaps in knowledge regarding Mpox diagnosis and management, stressing the need for targeted educational interventions. Similarly, the efficacy and acceptability study on an online Mpox curriculum (NCT05651581) found that structured educational modules significantly improved awareness and vaccine acceptance among high-risk populations. Vaccine-related studies, such as the evaluation of JYNNEOS (NCT05512949) and the Imvanex^®^ cohort study (NCT03745131), provided valuable data on immunogenicity and safety. These findings are instrumental in refining vaccination strategies, particularly concerning dose reduction to enhance vaccine accessibility without compromising efficacy.

At the mechanistic level, drugs used for the treatment of Mpox target the viral replication cycle. Tecovirimat, for instance, inhibits viral egress by targeting the F13 phospholipase. Structural studies reveal that tecovirimat acts as a molecular glue, forcing F13 homodimerization to block mature virion wrapping (Shamim et al., 2023; Vernuccio et al., 2025). Mutations in F13’s dimer interface (e.g., A284T, L314R) disrupt this interaction, conferring resistance by preventing drug-induced dimerization (Vernuccio et al., 2025). Beyond F13, tecovirimat also inhibits the VP37 protein, which mediates virion envelopment *via* interactions with host proteins TIP47 and Rab9, further limiting viral spread (Shamim et al., 2023). Alternative strategies target viral transcription and host pathways. Repurposed drugs like lumacaftor and imatinib bind the viral DNA-dependent RNA polymerase (DdRp), blocking RNA synthesis (Dutt et al., 2023), while Kaempferol-O-rhamnosides inhibit viral DNA-to-RNA transcription by interacting with RNA polymerase (Mashud et al., 2023). Host-directed therapies disrupt proviral signalling: Niclosamide and Sunitinib suppress PI3K-Akt and NF-κB pathways, reducing viral replication (Imani et al., 2024), and CDK4/6 inhibitors modulate Wnt/β-catenin and STAT3 signalling to counteract immune evasion (Imani et al., 2024; Alakunle et al., 2024). These dual approaches (direct antiviral activity and host pathway modulation) highlight the complexity of disrupting MPXV’s lifecycle and mitigating resistance risks (Table 2).

**TABLE 2:** Summary of mechanistic investigations on Mpox therapeutics, molecular targets, and drug interactions.

Drug/Compound	Primary molecular Target(s)	Mechanism of action	Experimental Model(s)	Noted drug–Drug interactions	Key findings
Tecovirimat (ST-246)	VP37 protein, F13 phospholipase	Inhibits virion envelopment and egress by stabilizing F13 homodimers, preventing mature virion formation	*In vitro*, animal models, structural studies	Minimal CYP3A4 metabolism; potential interactions with strong CYP3A4 inducers/inhibitors	Resistance associated with F13 mutations (A284T, L314R); high efficacy in animal models and early human data
Brincidofovir	Viral DNA polymerase	Lipid-conjugated prodrug of cidofovir; inhibits viral DNA synthesis	*In vitro*, smallpox animal models	Potential hepatotoxicity; additive nephrotoxicity with other renally-excreted drugs	Enhanced bioavailability vs cidofovir; lower nephrotoxicity; hepatotoxicity observed in some patients
Cidofovir	Viral DNA polymerase	Nucleotide analogue; inhibits viral DNA replication	*In vitro*, animal models	Severe nephrotoxicity risk; avoid with other nephrotoxic drugs	Requires probenecid and hydration; effective in severe cases but limited by toxicity
Lumacaftor	DNA-dependent RNA polymerase (DdRp)	Binds and inhibits DdRp, blocking viral RNA synthesis	*In vitro*, *in silico*	Not well characterized	Potential as repurposed drug; early-stage evidence only
Imatinib	Host Abl-family tyrosine kinases	Blocks virus entry and egress by inhibiting host kinase pathways	*In vitro*, *in silico*	Potential interactions with CYP3A4 substrates	Broad antiviral activity; host-targeted mechanism may reduce resistance risk
Kaempferol-O-rhamnosides	Viral RNA polymerase	Inhibits DNA-to-RNA transcription	In silico docking	Not well characterized	Natural compound with predicted inhibitory activity; needs experimental validation
Niclosamide	PI3K–Akt, NF-κB pathways	Inhibits host signaling pathways required for viral replication	*In vitro*, *in silico*	Interactions with CYP enzymes possible	Broad-spectrum host-targeted antiviral candidate
Sunitinib	PI3K–Akt, NF-κB pathways	Modulates host kinase signaling to suppress viral replication	*In vitro*, *in silico*	Multiple kinase-targeted drug interactions	Potential synergistic use with direct-acting antivirals

The mechanistic insights into the drug-virus interactions indicate the need for integrated strategies that target both viral and host factors in order to address resistance and improve therapeutic outcomes. One of the key targets for a therapeutic intervention lies in the optimization of viral targets. For example, F13 phospholipase inhibitors such as tecovirimat exert their inhibitory effect by stabilizing F13 homodimers; nonetheless, mutations such as A284T and L314R interfere with this interaction and thus bring about resistance (Vernuccio et al., 2025). Therefore, next-generation inhibitors will have to seek alternative binding sites or employ dual-target approaches to circumvent resistance mutations. Repurposed drugs, such as lumacaftor and imatinib, might also be useful in suppressing viral RNA synthesis through binding with DNA-dependent RNA polymerase (DdRp) (Dutt et al., 2023; Lu et al., 2023). Optimizing these compounds for greater specificity would yield better antiviral potency. Host-directed antiviral treatment also offers abundant promise. Drugs such as Niclosamide and Sunitinib inhibit PI3K-Akt and NF-κB signaling, thereby attenuating viral replication (Imani et al., 2024; Lu et al., 2023). Prioritizing kinase inhibitors with a broader spectrum of activity against proviral host factors should thus be advantageous in improving therapeutics. In addition, immunomodulation through CDK4/6 inhibitors and ERβ agonists counteracts immune evasion by remodelling Wnt/β-catenin and STAT3 pathways (Imani et al., 2024; Lu et al., 2023). Such combinations with direct antivirals could not only strengthen therapeutic benefits but also reduce the possibility of resistance.

## 6 Challenges and obstacles in drug development

Despite recent advances, the development of effective therapy for the treatment of Mpox faces significant challenges. Chief among these are drug resistance, the need for rapid deployment of therapeutics during outbreaks, and systemic barriers in the drug development pipeline. Among the therapeutics available for use on Mpox in the clinical setting, tecovirimat is first ranked. Although effective *in vitro* and animal models, point mutations in the F13L gene can render the drug less effective, raising concerns about resistance emergence under selective pressure (CDC, 2025). Due to the large DNA genomes and high-fidelity replication of orthopoxviruses, the mutation rate is lower than that of RNA viruses; however, resistance can still occur, especially with long monotherapy (Molteni et al., 2023). Recently, studies have revealed resistant strains of the MPXV to tecovirimat from clinical isolates. For example, genomic sequencing associated with the 2022–2023 outbreak in the United States revealed numerous mutations within the F13L gene, which were associated with resistance (Smith et al., 2023). Also, a cluster of cases distributed across five U.S. states between October 2023 and February 2024 was linked to MPXV strains carrying F13L mutations (N267del and A184T) for tecovirimat resistance (Gigante et al., 2024). Thus, Cross-resistance with anti-viral drugs having similar modes of action, such as Cidofovir or Brincidofovir, may further affect the options for treatment selection (Prévost et al., 2024). Combination therapy with agents having divergent mechanisms of action, such as the co-administration of tecovirimat with host-targeted agents such as mycophenolate mofetil (MMF) or N-myristoyltransferase inhibitor IMP-1088 against MPXV, has the potential to reduce the emergence of drug resistance (Witwit et al., 2025). Unfortunately, clinical data on these combination regimens are lacking, and there is a pressing need to evaluate their safety and efficacy.

Effective outbreak management depends on quick therapeutic intervention. Unfortunately, this is often slowed down by delays in getting regulatory approvals, logistical challenges, and limited production capabilities. With Mpox, these issues are even more pronounced due to its classification as a neglected tropical disease and the relatively small financial incentive for pharmaceutical companies (Rahim et al., 2024). In response to the COVID-19 pandemic, the Emergency Use Authorization (EUA) mechanism employed has also proven beneficial for Mpox. Regulatory bodies like the FDA and EMA have given conditional approval for tecovirimat (Rahim et al., 2024). However, limitations due to the reliance on animal models under the Animal Rule and limited efficacy data from clinical studies constrain the robustness of these authorizations (Rahi et al., 2023). Adaptive designs of clinical trials, including the use of platform trials or seamless phase transitions, are thus being introduced as possible solutions to speed up the evaluation and approval. However, requirements like coordination and real-time data monitoring systems is usually lacking in endemic settings (Ahmed et al., 2024). Ethical and logistical challenges due to global inequities in antiviral access are potential concerns (Danladi et al., 2024). Additionally, vulnerabilities in the supply chain, such as dependency on limited active pharmaceutical ingredient (API) suppliers, can stifle fast distribution when an emergency happens (Adak, 2024).

Preclinical research suffers from a lack of animal models and concerns about biosafety despite the availability of MPXV genome sequences. MPXV experimentation requiring BSL-3 or BSL-4 laboratory-level containment puts a constraint on the number of institutions capable of undertaking this research. Additionally, methods employed for evaluating surrogate viruses like vaccinia may at times hinder the accurate prediction of the therapeutic efficiency against MPXV, thereby piling on the dilemma of preclinical evaluation (Grajales and Kar, 2024). Additionally, biosafety concerns limit access to MPXV-positive serum samples, which are essential for the validation of the diagnostic and therapeutic techniques (Xiangjun et al., 2025). The investment in the Mpox drug development is strained. As a sporadic disease with limited commercial appeal, Mpox does not attract significant interest from the pharmaceutical industry. Most ongoing research is funded by public agencies or NGOs, which may be insufficient to drive large-scale clinical trials (Zumla et al., 2025). Without the continuous influx of funds, the enhancement of therapy and the formulation of vaccines are delayed, thus putting the preparedness for future outbreaks at risk. In addition to these, there is also the issue of the regulatory uncertainty that causes more challenges, especially in the low- and medium-income countries. The approval and distribution of the delivery of drugs are delayed due to fragmented regulatory frameworks and limited infrastructure for clinical trials (Sudarmaji et al., 2022). Moreover, it is a reality that the occurrence of such a lack of solidarity in the pursuit of health could be noted in many cases. Though there are a few cases of collaboration, such as the WHO R&D Blueprint collaboration framework, powerful forces still work against such development of research efforts. The opposition to intellectual property rights, an array of researched fragments, and unstandardized trial protocols are all hindrances to progress (Aggarwal et al., 2023; Cambaza, 2025). The bridge to this gap is a combination of public and private skills, the efforts of consortia, and open access to data, where the public and private sectors work hand in hand towards the common objective, and in the same country, the smartness of research combines itself into one file.

## 7 Next-generation therapies

The resurgence of Mpox as a global health concern has intensified the search for effective therapeutic strategies. Traditional treatments have focused on symptom management and the repurposing of existing antivirals as the major approaches. However, the limitations of these very approaches have spurred the exploration of next-generation therapies (Table 3). Given the success of mRNA technology in COVID-19 vaccines, researchers have started to study various mRNA-based vaccines for Mpox. Moderna’s mRNA vaccine candidate, namely mRNA-1769, was successful in preventing the disease in preclinical studies, displaying a reduction in disease severity and duration in macaque models (Cotter et al., 2024). Macaques that received the mRNA vaccine exhibited fewer symptoms, including weight loss and lesions, and had lower viral loads and a shorter disease duration compared to those that received the MVA vaccine (Mucker et al., 2024). This approach allows for rapid vaccine development and scalability, crucial during outbreak scenarios.

**TABLE 3:** Selected emerging therapeutic technologies for Mpox.

Technology	Mechanism/Benefit	Examples	Development status
mRNA Vaccines	Induce adaptive immunity through synthetic mRNA encoding viral antigens	Moderna’s mRNA-1769	Preclinical in non-human primates
Monoclonal Antibodies	Neutralize virus by targeting specific epitopes on MPXV proteins	MPV-A1, MPV-B5 (experimental candidates)	Experimental phase
Nanoparticle-Based Delivery	Enhance drug/vaccine stability and targeted delivery	Lipid-based mRNA carriers, silver nanoparticles	Under active research
In Silico Drug Repurposing	Use AI tools to identify antiviral properties in known compounds	Tecovirimat analogues, curcumin-like compounds	Computational/early *in vitro*

Nanomedicine offers innovative solutions to circumvent challenges posed by targeted drug delivery for enhanced therapeutic effects. Nanoparticles can be designed to increase the stability and bioavailability of antiviral agents and enable their targeted delivery to infected cells. Lipid nanoparticulate systems could be used to encapsulate mRNA *in vitro*, thus increasing their delivery efficiencies and immune response (Swetha et al., 2023). Metal-based nanoparticles act by blocking viral entry and replication: gold and silver nanoparticles exhibit such antiviral properties (Maduray and Parboosing, 2020). This places nanotechnology as a promising option to further develop Mpox therapeutics with better efficacy and safety profiles. Various computational approaches were earlier used to identify potential antiviral compounds against MPXV using *in silico* docking methods. High-throughput virtual screening has identified many compounds with potential antiviral activity against Mpox. Some drugs, including fludarabine, naldemedine, and saquinavir, have shown a promising outcome *in silico*, hence opening new avenues for drug development (Shamim et al., 2023). Repurposing existing drugs can thus serve as a cost-effective and time-saving means to developing newer treatments. Imatinib, Conivaptan, Lumacaftor, Betulinic acid, and Fluspirilene are examples of compounds identified as prospective agents with antiviral activity against orthopoxviruses (Dutt et al., 2023; Khan et al., 2023). This strategy offers a cost-effective and time-efficient pathway to identify promising therapeutic candidates for further experimental validation.

These prospects serve as disease-reducing opportunities, enhancing better prophylaxis and preparation for future outbreaks through rapid-response platforms. Future perspectives for Mpox treatment are oriented toward employing emerging technologies alongside conventional therapeutics. A combination of drugs for different life-cycle stages of the virus can increase the effectiveness and decrease the resistance. For instance, combining tecovirimat with nucleoside analogs like cidofovir could produce synergistic effects by inhibiting both viral egress and nucleic acid synthesis, respectively (Akazawa et al., 2023). Through genomics and proteomics advancements, personalized treatment strategies could be developed through the identification of relevant viral and host features to optimize therapeutic choices and to be more selective and rigorous (Sahu et al., 2023).

## 8 Interactions with comorbid treatments

**FIGURE 3:**
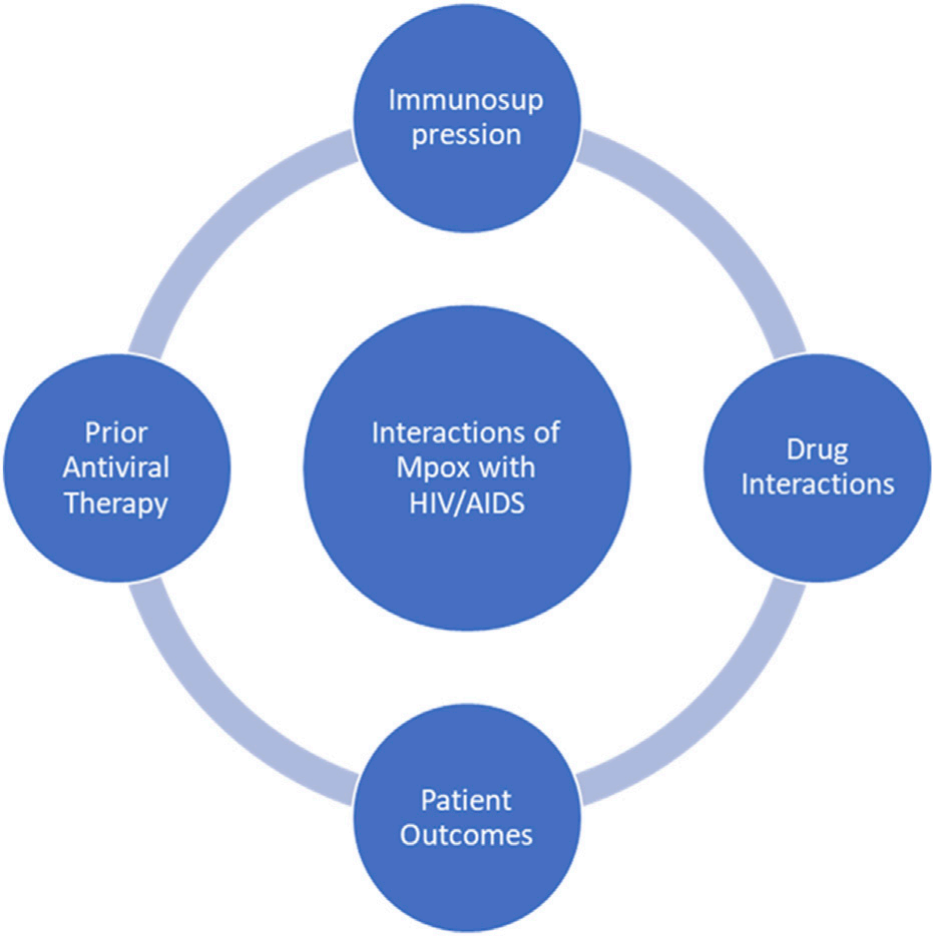
A circular illustrating of the interplay between mpox and HIV/AIDS.

Mpox has demonstrated a complex clinical course in patients with immunocompromised conditions (Figure 3), such as HIV/AIDS, potentially resulting in large lesions, bacterial superinfections, and prolonged periods of disease (Ahmed et al., 2023). As the outbreaks become more frequent and widespread globally, growing concern has arisen regarding how comorbidities and prior antiviral therapies influence Mpox pathogenesis, treatment efficacy and prognosis (Saldana et al., 2023). Understanding these dynamics is very important for the development of treatment algorithms that will reduce mortality and promote optimal allocation of resources in outbreak settings. The immunological deficits in untreated or advanced HIV infection considerably predispose to a severe form of Mpox manifestation. Studies from the 2022–2023 outbreaks showed that, globally, persons living with HIV (PLWH) accounted for about 38%–50% of confirmed cases of Mpox, with a disproportionate share of morbidity and mortality occurring among those with CD4^+^ T-cell counts <200 cells/mm^3^ (Mitjà et al., 2023; Thornhill et al., 2022). In these populations, Mpox may present atypically as widespread necrotizing lesions associated with bacterial superinfections and extended viral shedding, inciting a possible greater risk of enduring infectiousness and community transmission (Miller et al., 2022; Ogoina et al., 2020).

ART status substantially influences the Mpox disease course. Most people with well-controlled HIV infection often have clinical courses similar to those of negative patients. However, those who have suboptimal ART or are not on ART frequently suffer from worsened Mpox, with a mortality rate of 27%, compared to <1% in immunocompetent individuals (Saldana et al., 2023; Miller et al., 2022; Bender Ignacio et al., 2025). HIV and Mpox co-infected patients often need concurrent administration of ART and an antiviral. Tecovirimat, the most popular antiviral choice for Mpox, blocks the VP37 protein, which is essential for viral egress (Hudu et al., 2023). The drug, which is generally well tolerated, has been known to be metabolized by CYP3A4; therefore, a concern was raised against its concurrent use with protease inhibitors like ritonavir or ritonavir-boosted darunavir, given that these drugs are substrates and inhibitors of CYP3A4. Such interactions may call for dose adjustments and/or intensified pharmacovigilance. Brincidofovir has the potential to elevate hepatic enzymes, and hence, caution should be exercised, particularly in those patients on hepatotoxic ART regimens like nevirapine (Shamim et al., 2023).

The concern of IRIS (Immune Reconstitution Inflammatory Syndrome) has thrived for years when initiating ART in the presence of active opportunistic infections. More recent evidence refutes the scenario of early ART in Mpox worsening inflammatory pathology or the outcome (Rodríguez-Aldama et al., 2024). Conversely, other studies suggest that those with delayed ART in the setting of Mpox suffer longer illness and more complications (Saldana et al., 2023). *S. aureus*, *C. albicans*, and various herpesviruses frequently present as co-infections complicating MPX management in immunocompromised persons. Therefore, in these patients, MPX may serve as a syndemic co-factor, aggravating the pre-existing morbidity associated with HIV/AIDS (Yinka-Ogunleye et al., 2023). Among 81 HIV-positive Mpox patients hospitalized in sub-Saharan Africa, 47% developed sepsis, and 19% required intensive care. The median time to lesion resolution was 18 days for ART-naïve vs 11 days in those on ART ≥6 months. Mortality was strongly correlated with baseline CD4^+^ <50 cells/mm^3^ and lack of ART adherence (Mitjà et al., 2023). The clinical recommendations for the management of Mpox in PLWH are shown in Table 4.

**TABLE 4:** Clinical management recommendations for Mpox in PLWH.

Clinical scenario	Recommendation
ART-naïve with Mpox infection	Initiate ART and tecovirimat simultaneously; monitor for IRIS and liver toxicity
On ART with suppressed viral load	Treat Mpox with standard antivirals; anticipate normal recovery timeline
Advanced HIV (CD4^+^ <200 cells/mm^3^)	Consider hospitalization; treat with tecovirimat ± cidofovir; manage complications aggressively
Co-infections present	Administer broad-spectrum antibiotics or antifungals as indicated

Patients with untreated HIV infection should initiate ART and tecovirimat simultaneously. Monitoring for IRIS and liver toxicity is critical during this phase. Early administration of tecovirimat has been shown to reduce viral replication by over 90% within 3–5 days and shorten the time to viral clearance by approximately 6 days if initiated at symptom onset (Nguyen et al., 2023). However, IRIS may complicate recovery, necessitating close observation. For patients on ART with a suppressed viral load, standard antiviral treatment for mpox, such as tecovirimat, is recommended. These individuals typically experience a normal recovery timeline without significant complications (Ivanov et al., 2023). Patients with advanced HIV and low CD4 counts (<200 cells/mm^3^) require hospitalization due to the risk of severe complications. Treatment should include tecovirimat ± cidofovir to manage Mpox aggressively. Cidofovir inhibits DNA polymerase, disrupting viral replication, while tecovirimat targets viral egress (Ivanov et al., 2023). Severe cases often present with prolonged disease courses and destructive lesions, as documented in advanced HIV patients treated compassionately with tecovirimat (Duani et al., 2024). In cases of co-infections alongside Mpox, broad-spectrum antibiotics or antifungals should be administered as indicated. For example, co-infections like syphilis or bacterial superinfection may necessitate tailored antimicrobial regimens (Duani et al., 2024). Managing these co-infections is essential to prevent further complications during Mpox treatment.

## 9 Expert opinion and future perspective

With the ever-increasing immunosuppression of our planet and the need of the global health community to face re-emerging zoonotic infections, Mpox is a burning example of the intertwinement between how viral evolution evolves and how our preparedness to treat an infection and equity of care also evolve. Even though tecovirimat and brincidofovir have been developed and employed under compassionate use, implementation has been mostly reactive and little infrastructure exists in endemic areas to accommodate routine clinical use. Among experts, the concern that has emerged most urgently is the excessive reliance on a very small number of repurposed antivirals, including tecovirimat, without clinical trial data that evidence the medications’ efficacy or safety over the long term in a wide array of patients. Future readiness efforts must prioritise the diversification of the Mpox therapy portfolio. This will include the acceleration of research on next-generation antivirals, including those that act on multiple stages of the Mpox viral lifecycle and so reduce the likelihood of resistance. The increasing risk of the emergence of tecovirimat-resistant strains of the virus has been making a case for dual-action or synergistic therapies that not only include an agent like cidofovir but also host-directed therapies. Pharmacokinetically optimized formulations for oral and pediatric administration should also be exploited in drug development for achieving broader and more equitable access. Conceptually, this can potentially change how we deal with future outbreaks by incorporating mRNA vaccine platforms and nanotechnology-based delivery systems in Mpox preparedness plans. The deployability and scalability of these technologies, as we saw during the context of COVID-19, are promising. But much depends on proactive investment, cross-border regulatory harmonization, and fairer intellectual property policies.

A synergy with an interdisciplinary “One Health” approach, taking into account the ecological and socioeconomic determinants of Mpox outbreaks, is also essential. Remediating the root causes of zoonotic spillovers, including deforestation, wildlife trade, and anaemic veterinary surveillance, should be considered part and parcel of therapeutic planning. Clinically, the future of Mpox management will rely on precision medicine strategies that are guided by genomics, resistance profiling, and patient-specific considerations such as the presence of comorbidities, notably HIV infection. Artificial intelligence and machine learning have the potential to be transformational in antiviral selection, resistance pattern prediction, and outbreak trajectory modelling.

Ultimately, long-term financing and global collaboration will be essential. Even the most basic therapeutic options remain inaccessible for many low- and middle-income countries due to financial, logistical, and political constraints. The global health community must advocate for strategies that facilitate the swift deployment of new treatments in vulnerable regions, such as regional manufacturing capacity, pooled procurement, and tiered pricing. While some significant pharmacological advancements have been made in Mpox treatment, current efforts should focus on developing progressive, lasting, and universal solutions. The experience of Mpox should guide not only future outbreak responses but also the criteria by which we evaluate drug development, access, and global health solidarity amidst emerging infectious diseases.

## 10 Conclusion

Mpox becomes an urgent challenge in infectious disease control without specific antiviral drugs and under drug-resistant strains. Existing treatments are mainly antiviral agents repurposed with different levels of effectiveness and safety. Advances in molecular pharmacology, such as host-pathway modulators and mRNA vaccine platforms, provide new hope. But fair access, regulatory alignment and continued R&D investment are crucial. A multifaceted approach of pharmacology, surveillance and rapid diagnosis is essential to minimize the global Mpox burden and for pandemic preparedness.
